# Retrospective study of detecting oesophageal injuries post neck trauma: CTA versus fluoroscopy

**DOI:** 10.4102/sajr.v28i1.2930

**Published:** 2024-09-09

**Authors:** Audrey R. Rumhumha, Nicholas Christofides, Pravani Moodley

**Affiliations:** 1 Department of Diagnostic Radiology, Faculty of Health Sciences, School of Clinical Medicine, University of the Witwatersrand, Johannesburg, South Africa; 2 Department of Family Medicine, Division of Emergency Medicine, University of the Witwatersrand, Johannesburg, South Africa

**Keywords:** oesophageal injury, penetrating neck trauma, CTA, fluoroscopy, fluoroscopic oesophagography

## Abstract

**Background:**

Timely detection of oesophageal injuries post-penetrating neck trauma is imperative because of the associated high morbidity and mortality. Patients commonly undergo both CT angiography (CTA) and contrast swallow studies (fluoroscopic oesophagography) when oesophageal injury is suspected.

**Objectives:**

To determine the radiological findings of oesophageal injury after penetrating neck trauma comparing CTA and fluoroscopic oesophagography at a single tertiary centre.

**Method:**

The study retrospectively reviewed the data from CTA and fluoroscopic oesophagography reports of patients suspected of oesophageal injuries secondary to penetrating neck trauma at a tertiary hospital in South Africa from January 2018 to December 2022.

**Results:**

A total of 76 records were reviewed. The mean age for the participants was 31.5 years, ranging from 0.75–66 years. In this study 6/76 (8%) patients had confirmed oesophageal injury on fluoroscopy, which is considered the gold standard. The majority of penetrating neck injuries were in the 20–29 year age group, with 33/76 (43%) injuries. Stab wounds as the mechanism of injury accounted for 57/76 (75%). Dysphagia was experienced by 10/76 (13%) of those who had injuries. Zone I injuries accounted for 33/76 (43%) of the injuries.

**Conclusion:**

The incidence of oesophageal injuries secondary to penetrating neck injuries is comparable to previous studies. This study determined that CTA has a high sensitivity but low specificity.

**Contribution:**

Fluoroscopic oesophagography should, therefore, be performed in patients who have an abnormal CTA coupled with clinical signs and symptoms of oesophageal injury.

## Introduction

Oesophageal injury resulting from penetrating neck trauma carries significant risk of complications and is associated with high morbidity and mortality.^1^ Detecting these injuries promptly is crucial as swift identification and management play a role in achieving a more successful outcome. Delay in intervention is associated with a significantly elevated mortality rate.^2,3,4^

Trauma in South Africa has been characterised as a severe and widespread issue, often referred to as a ‘malignant epidemic’, placing a substantial strain on the already limited resources of trauma centres across the nation.^5^ The burden of trauma in South Africa primarily stems from a mix of interpersonal violence and motor vehicle accidents (MVAs),^6^ contributing to the high prevalence of penetrating neck injuries (PNIs) seen in trauma centres in the country.^7^

Chris Hani Baragwanath Academic Hospital (CHBAH) plays a significant role in managing patients with penetrating neck, chest and abdominal injuries,^3^ with approximately 380 such cases treated on a monthly basis. Additionally, the hospital typically accommodates over 150 trauma inpatients at any given time, reflecting the substantial impact and volume of trauma cases managed at CHBAH.^6^

The defining feature of a significant PNI is the breach of the platysma layer in the neck.^8^ Penetrating neck injuries are prevalent in urban trauma settings globally, with South Africa predominantly experiencing PNIs secondary to stab wounds, although gunshot injuries account for 11%.^9^ Similarly, in the United States, head and neck penetrating traumas resulting from stabs and gunshots constitute a significant portion of emergency department presentations.^10^

The complex interplay of potential neurovascular and aerodigestive injuries in such cases presents clinicians with challenging diagnostic scenarios, emphasising the importance of accurate and timely decision-making in the management of these injuries. While oesophageal trauma is relatively uncommon, occurring in only 0.9% – 6.6% of cases of PNIs,^11,12^ it stands out as the most frequently overlooked injury in this region. A delayed diagnosis of oesophageal trauma can lead to significant complications,^13^ with mortality rates reaching up to 50% and risks including mediastinitis, abscess development and septicaemia.^14^

Even in busy urban trauma centres, penetrating oesophageal injuries are an unusual entity.^10^ The reason for the rarity of injury is related to the anatomically nested position of the oesophagus. There is a paucity of data regarding purely traumatic oesophageal injuries because many studies combine iatrogenic and medical-related oesophageal injuries (e.g. Boerhave’s) with traumatic injuries.^14^ Patients in the medical facilities are often imaged using both CT angiography (CTA) and contrast swallow studies (fluoroscopic oesophagography) when there is concern for an oesophageal injury.^13^

Multidetector computed tomography (MDCT) has become the method of choice and is the ‘workhorse’ in imaging of patients who present with trauma. It is an excellent tool for the detection of vascular injury, which is seen in up to 25% of penetrating wounds.^8^ However, there is limited knowledge of its accuracy for oesophageal injuries.^14^

The CT findings of oesophageal injury include oesophageal wall thickening, peri-oesophageal gas and fluid collection, mediastinal fluid collection, mediastinal inflammation and a focal oesophageal wall defect.^9^ When oesophageal injury is suspected, fluoroscopic oesophagography and endoscopy are indicated. Suspected oesophageal perforation is recognised by the American College of Radiology (ACR) as an indication for fluoroscopic oesophagography (ACR appropriateness criteria).^15^

Despite recommendations from the ACR, approximately 10% – 12% of oesophageal perforations may be missed on fluoroscopic oesophagography when using barium and 22% – 50% of cases may be missed on fluoroscopic oesophagography when using water-soluble contrast material alone.^16^ Fluoroscopic oesophagography also has several other limitations. The examination requires adequate radiology staffing, which is often limited after hours, transportation to the fluoroscopy unit, patient cooperation and positioning, appropriate level of consciousness, and ability to swallow, any of which may be compromised in an acutely unwell patient. In addition, fluoroscopic oesophagography poses a risk for aspiration or pulmonary oedema.^16^

CT avoids many of the limitations associated with fluoroscopic oesophagography and has the advantage of providing further information that may guide decisions on surgical management.^17^ CT is more readily available and has rapid image acquisition ability. The current standard practice, which is based on older studies in the surgical literature, is to obtain a fluoroscopic oesophagogram for suspected oesophageal perforation regardless of whether a CT scan has been performed.^18^

Although oesophageal injuries are infrequent, they are linked to unfavourable patient outcomes.^19^ Timely investigation and intervention for possible oesophageal injuries is therefore critical. This research aimed to primarily determine the incidence of oesophageal injuries resulting from penetrating neck trauma, detailing the clinical presentation and demographic data of these patients. The secondary objectives involved assessing the diagnostic accuracy of CTA in comparison to the reference standard of fluoroscopic contrast swallow studies, and identify radiological indicators of oesophageal injury observed on both CTA and fluoroscopy. Moreover, the study aimed to evaluate whether the combined use of CTA and fluoroscopic oesophagography offers enhanced sensitivity in detecting oesophageal injuries compared to using either modality independently.

## Research methods and design

This was a retrospective cross-sectional review of CTA and fluoroscopic oesophagography reports of patients with suspected oesophageal injury secondary to penetrating neck trauma. Reports of 97 patients were identified from the hospital picture archiving communication system (PACS) from 01 January 2018 to 31 December 2022, and analysed.

A sample size of 123 records was determined, taking into consideration that 8.7% of PNIs involve oesophageal injury with a 95% confidence level and 80% power to ensure a statistically significant study. By employing Equation 1:

$$\text{Sample size }n=\left(\text{DEFF}\times \text{N}p[1-p]\right)/\left({\text{d}}^{2}/{\text{Z}}_{1-\alpha /2}^{2}\times [N-1]+p\times [1-p]\right)$$ [Eqn 1]

Our sample size of 76 yielded a confidence interval ranging from 80% to 90%.

Computed tomography angiography studies were performed on a multidetector CT scanner at CHBAH. Eighty millilitres of non-ionic contrast material (omnipaque) was injected intravenously. Scanning was initiated automatically by the machine when an adequate bolus of contrast reached the aorta (bolus tracking technique) and images were acquired in transverse section.

Fluoroscopic swallow examinations were performed on CHBAH digital fluoroscopic machines. The contrast swallow examination was performed by asking the patient to swallow 50–100 mL of non-ionic contrast material (omnipaque). Images were acquired in the frontal and lateral projections. Additional images were sometimes acquired with the injury site being placed in a dependent position. The principal investigator was primarily responsible for data collection and data analysis, supported by a biostatistician.

Patients of all ages, including adults and paediatric patients, who presented with suspected oesophageal injury following penetrating neck trauma and had both CTA and a fluoroscopic swallow study within 72 h of each other, regardless of the order in which the examinations were conducted were included in the study. The exclusion criteria consisted of patients with insufficient information to fulfil the primary objectives, individuals with a history of prior oesophageal surgery preceding the CTA or fluoroscopy investigations, patients who had more than a 3-day gap between the CTA and fluoroscopic examination, and those who sustained a mechanism of injury other than penetrating neck trauma. These criteria aimed to ensure a focused and consistent study population for the research objectives.

Computed tomography angiography reports were either prepared by registrars and confirmed by a consultant or directly reported and validated by a consultant. Similarly, all fluoroscopic oesophagography procedures were conducted and interpreted by a registrar within the department, under the supervisory guidance and support of a consultant. Data were extracted from CTA and fluoroscopy reports and populated onto an Excel spreadsheet. The data included patients’ demographics (age and gender), clinical signs and symptoms, mechanism of injury, neck zone of injury, CTA findings, including other associated injuries, and fluoroscopic findings. A positive result was defined according to a predefined range of abnormalities.

The criteria for a positive CTA diagnosis: Concluding remark in the final report indicating the presence of oesophageal perforation. The presence of any of the following: Air in deep or superficial neck spaces, air locules around the oesophagus, pneumomediastinum, pneumothorax. Deep neck spaces were defined as deep to cervical fascia and superficial neck spaces as superficial to it. Presence of signs associated with the trajectory of the wound: Tract into or through the aerodigestive system, tract violating the deep neck spaces as well as fluid in the deep neck spaces, wall defect and thickened or irregular wall.^1^ Injuries were classified according to zonal anatomy. Location of zone was allocated as described by the referring clinician into the three anatomical standard zones of the neck as defined by Roon’s classification. Zone I, from the sternal notch to the cricoid cartilage; Zone II, from the angle of the mandible to the cricoid cartilage; and Zone III, from the base of skull to the angle of the mandible.^20^ The criteria for a positive fluoroscopy swallow diagnosis: Final report interpretation of oesophageal perforation, presence of extraluminal contrast material from the oesophagus.

Computed tomography angiography and fluoroscopic oesophagography findings were compared and analysed. Fluoroscopic oesophagography was considered the standard reference for the presence of oesophageal injury. True positives required the correct definition by type and location of injury.

### Statistical analysis

Data were entered into a de-identified Excel sheet from the data collection sheets. The data were imported into Stata Version 15 (Stata Corp) for further analysis. Different commands assisted in cleaning the data. Data cleaning processes included checking for duplicates, missing values, recording and categorising variables. Descriptive statistics was conducted. Categorical variables were presented as frequency and percentages (mechanism, signs, symptoms, etc.). The continuous variables such as age were presented as mean ± standard deviation or median and interquartile range (if not normally distributed). The trend in different variables was presented graphically to show the distribution. Orange software was used to analyse the frequency of multiple text findings for CTA findings and additional sites of injury. Missing information was checked on each variable. Association between fluoroscopy and categorical variables such as group mechanism was assessed using the Pearson’s Chi-Square or Fisher’s exact test. Statistically significant correlation was set at *p*-value < 0.05.

### Ethical considerations

The study was approved by the Human Research Ethics Committee of the University of the Witwatersrand (certificate number M230138). Participants’ consent was not sought as this was a retrospective record review; to maintain strict anonymity, no personally identifiable information was recorded.

## Results

Over a period of 5 years, from January 2018 to December 2022, our search strategy yielded a total of 97 patients. From these, 21 cases were excluded for the following reasons: More than 72 h lapsed between the CTA and fluoroscopy studies, with a lag period of up to 21 days in one case. There were incomplete or missing reports from the database. One patient had emergent oesophageal surgery before imaging was performed. Four patients were found to have a mechanism of injury not related to penetrating neck trauma: One foreign body ingestion, two caustic ingestions and one hanging. The final study cohort comprised 76 patients. All imaging was of diagnostic quality and had complete reports with supporting clinical data available on PACS for review.

There were variations in time delays between the CTA and fluoroscopy studies ranging from 4 h up to 72 h. All but one patient had CTA before fluoroscopy. The mean age for the participants was 31.5 years with a range of 0.75–66 years. The median age for females was 24 years with a range of 21–30 years, while for males it was 30 (26–37) years.

In this study a total of 9/76 (12%) patients had evidence of injury to the upper gastrointestinal tract structures and were recorded as having positive fluoroscopic findings on oesophagography. Of these, 6 had oeosophageal injury and 3 had hypopharyngeal injury. Table 1 depicts the baseline characteristics of patients with a PNI, divided into those with and without confirmed pharyngo-oesophageal injury.

**TABLE 1:** Demographic and baseline characteristics of patients with and without pharyngo-oesophageal injury.

Category	Total		Positive		Negative		*p*
	*n*	%	*n*	%	*n*	%	
**Age**	-	-	-	-	-	-	1.000
< 20	5	7	0	0	5	7	-
20–29	33	43	5	56	28	42	-
30–39	25	33	3	33	22	33	-
40–49	7	9	1	11	6	9	-
50–59	2	3	0	0	2	3	-
60+	4	5	0	0	4	6	-
**Sex**	-	-	-	-	-	-	1.000
Female	9	12	1	11	8	12	-
Male	67	88	8	88	59	88	-
**Mechanism**	-	-	-	-	-	-	0.212
MVA	2	3	1	11	1	1	-
GSW	16	21	3	33	13	19	-
GSW (pellet gun)	1	1	0	0	1	1	-
SW	57	75	5	56	52	79	-
**Signs and symptoms**	-	-	-	-	-	-	0.321
Emphysema	5	7	1	11	4	6	-
Haemoptysis	2	3	0	0	2	3	-
Multiple signs	5	7	1	11	4	6	-
Nil signs	4	5	0	0	4	6	-
Open wound	60	78	7	78	53	79	-
Dysphagia	10	13	1	11	9	13	-
Dyspnoea	3	4	0	0	3	4	-
Hoarse voice	1	1	0	0	1	1	-
Multiple symptoms	2	3	1	11	1	1	-
Nil symptoms	54	71	5	56	49	73	-
Odynophagia	6	8	2	22	4	6	-
**Zone of injury**	-	-	-	-	-	-	0.184
I	33	43	3	33	30	45	-
II	30	40	3	33	27	40	-
III	5	7	0	0	5	7	-
I and II	1	1	0	0	1	1	-
I and III	7	9	3	33	4	6	-

MVA, motor vehicle accidents; GSW, gun shot wound; SW, stab wound; ECA, external carotid artery.

### Mechanism of injury

Of the 76 patients, most injuries were related to stab wounds (75%), followed by gunshot wounds. Two patients were involved in motor vehicle accidents resulting in penetrating injuries from blast injuries, and one patient was injured by a pellet gun. Among patients with pharyngo-oesophageal injury, the majority were from stab wounds, followed by gunshot wounds and a smaller proportion from a motor vehicle accident.

### Site of injury

**FIGURE 1:**
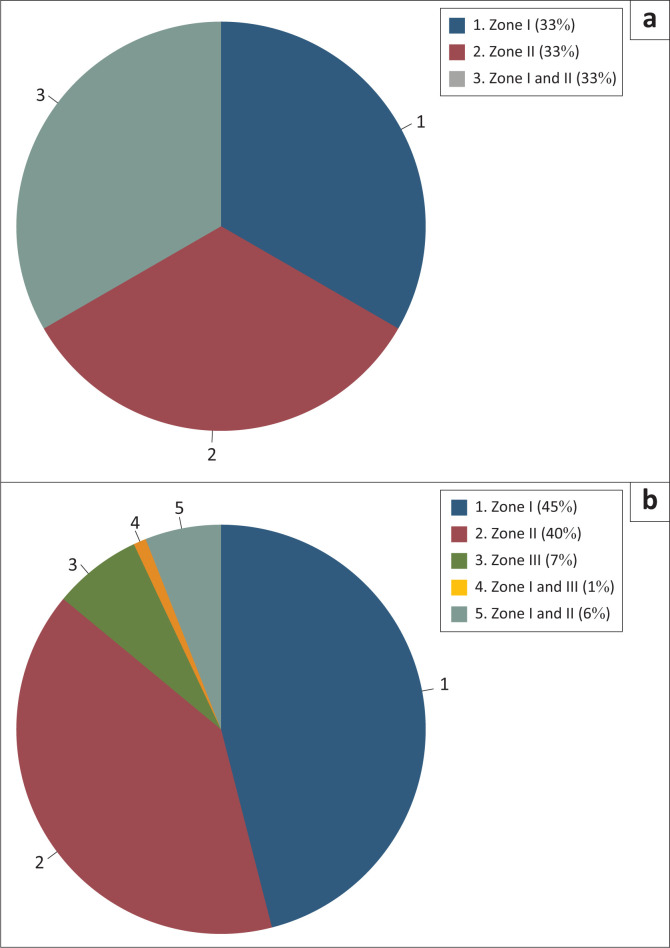
Distribution of sites of injury by zone in those (a) with and (b) without pharyngo-oesophageal injury.

Zone I accounted for most injuries, followed by Zone II, with the least number in Zone III. Among the patients with pharyngo-oesophageal injury, one-third had injuries in Zone I, another third in Zone II, and the remaining across multiple zones. The distribution of injury zones in patients with and without pharyngo-oesophageal injury is depicted in Figure 1.

### Clinical signs and symptoms

The request form indicated various clinical signs for suspected oesophageal injury. Among 76 patients, most (78%) had open wounds in the neck, other signs included were: emphysema, haemoptysis, while others had multiple findings such as open wounds with neck haematoma or emphysema. In those with confirmed pharyngo-oesophageal injury, the majority presented with open wounds, while others displayed emphysema, or had both an open wound and emphysema.

Clinical symptoms reported included dysphagia, odynophagia, dyspnoea, with some patients experiencing multiple symptoms. A large number of patients reported no symptoms. Among patients with pharyngo-oesophageal injury, the most common symptom reported was odynophagia, followed by dysphagia, with more than half reporting no symptoms.

### Radiological findings

#### Computed tomography angiography

A multitude of signs were demonstrated at CTA, most of which were indirect signs of oesophageal injury with only one patient having a direct sign of injury – a focal wall defect in the oesophagus. The presence of any of these signs in conjunction with a suspicious history and examination was flagged as positive and requiring further workup with fluoroscopic oesophagography. The CTA signs were categorised into air-associated, tract-associated, and conventional signs with the most frequently observed signs being related to air.

**FIGURE 2:**
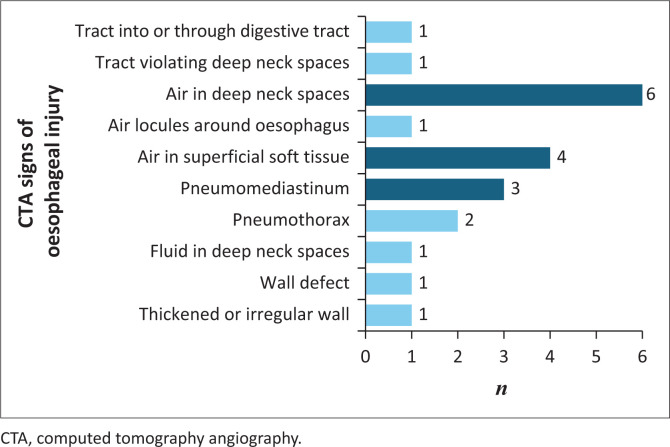
Bar graph depicting CT signs in patients with confirmed injury.

**FIGURE 3:**
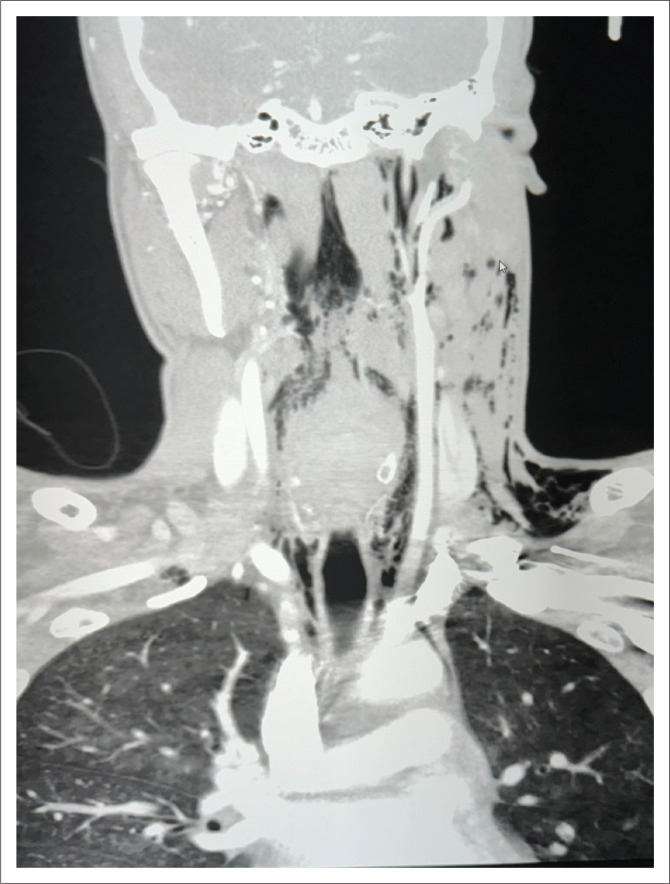
Coronal CT scan image of the neck and upper chest demonstrating superficial and deep surgical emphysema post penetrating neck trauma, suggestive of oesophageal injury.

In patients with confirmed pharyngo-oesophageal injury, the highest frequency was of the air-associated signs. Refer to Figure 2 for details. Also, refer to Figure 3, which illustrates the superficial and deep surgical emphysema on CTA neck imaging.

#### Fluoroscopy

**FIGURE 4:**
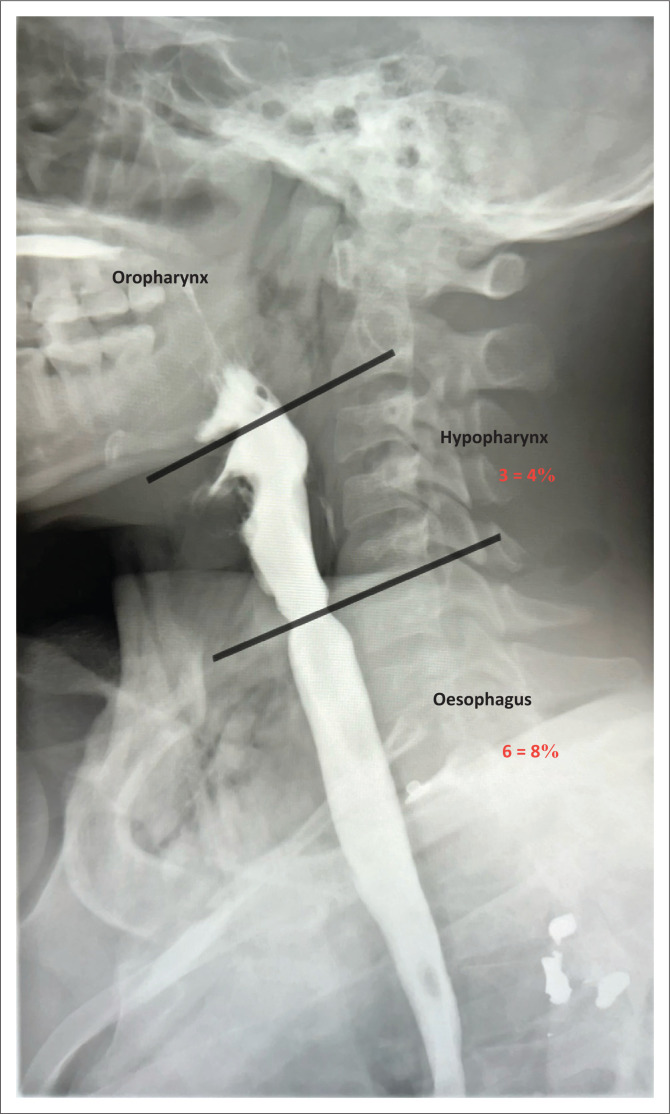
Anatomical divisions of the upper digestive tract and incidence of injuries in the hypopharynx and oesophagus. Extraluminal contrast is present posterior to the oesophagus at the level of the C5-C6 vertebrae in keeping with cervical oesophageal injury.

**FIGURE 5:**
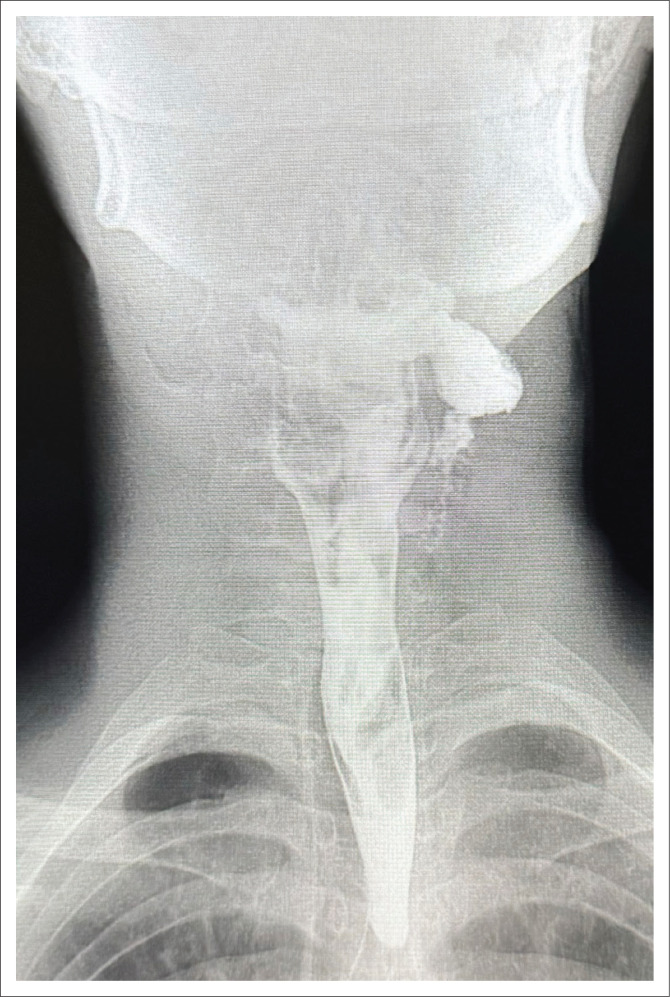
Fluoroscopic oesophagogram demonstrating frank extravasation of contrast material from the hypopharynx consistent with hypopharyngeal perforation.

The presence of extraluminal contrast signified the presence of injury within the upper digestive tract. The findings were further classified into pharyngeal injury and oesophageal injury based on the anatomic location of the extraluminal contrast (Figure 4 and Figure 5).

### Associated injuries

The most frequently observed injuries related to PNI were thoracic injuries, noted in 25 cases. Within this group, haemothorax was present in nine patients. Neck injuries, which included injuries to the thyroid lobe, hyoid bone fractures, and injuries to the larynx, trachea, and strap muscles also occurred. Additionally, there were cases of craniofacial injuries and vertebral injuries, as well as three incidents of vascular injuries.

In patients with pharyngo-oesophageal injury, the most common associated injuries were also thoracic injuries, specifically one case each of haemothorax, rib fracture and clavicle fracture. This was followed by vertebral injuries, and least commonly neck, craniofacial, and vascular injuries (Table 2 and Table 3).

**TABLE 2:** The frequency of associated injuries in patients presenting with penetrating neck injuries.

Associated injuries	Specific injury location	*n*
Thoracic	9 haemothoraces, 2 scapular fractures, 1 sternal fracture, 3 clavicular fractures, 4 rib fractures, 6 pulmonary lacerations and contusions	25
Neck	3 hyoid bone fractures, 9 thyroid lobe injuries, 2 laryngeal injuries, 2 tracheal injuries, 1 strap muscle injury	17
Craniofacial	6 mandibular fractures	6
Vertebral	5 vertebral injuries (2 C-spine, 3 T-spine), 1 pneumorrhacchis	6
Vascular	2 vertebral artery, 1 ECA injury	3

ECA, external carotid artery.

**TABLE 3:** Associated concomitant injuries in patients with confirmed pharyngo-oesophageal injury.

Associated injuries	Specific injury location	*n*
Thoracic	1 haemothorax, 1 rib fracture, 1 clavicle fracture	3
Neck	1 hyoid bone fracture	1
Craniofacial	1 mandible fracture	1
Vertebral	2 vertebral (1 C-spine, 1 T-spine)	2
Vertebral	2 vertebral (1 C-spine, 1 T-spine)	1

### Computed tomography angiography versus fluoroscopy

The overall sensitivity and specificity for CTA to detect oesophageal injury were 100% and 0%, respectively; with a positive predictive value (PPV) of 11.8% and a negative predictive value (NPV) of 0%. Computed tomography angiography had many false positives. Among the 70 patients showing false positive findings for oesophageal injury, 92% exhibited at least one abnormal finding on their CTA scans. The CTA accuracy was 11.8%.

There was 12% (95% confidence interval [CI] 0.04–0.20) agreement between fluoroscopy and CTA. In terms of the agreement coefficient of Kappa and Gwet, the values were 0.0000 (95% CI -0.00 – 0.00) and −0.7388 (95% CI -0.9155 – 0.5620), respectively. On the benchmark scale, the agreement coefficients were poor.

## Discussion

Penetrating injury to the cervical oesophagus is an uncommon injury because of its central and protected location, occurring in 3% – 6% of neck injuries.^11,12^ As a result of its low prevalence, numerous previous studies have been limited in size and of these studies, a large number combine the aetiology of the oesophageal injuries from various causes.^21^ Comparison to previous reports was thus limited as the focus of this study was specifically on injuries secondary to penetrating neck trauma.

Of the digestive tract injuries, injuries to the pharynx and cervical oesophagus are more common than injuries to the thoracic oesophagus, which is relatively well embedded in and protected by the thoracic cavity.^22^ In this single-centre, retrospective study within a tertiary hospital in Gauteng, pharyngo-oesophageal injury occurred in 12% of patients, and oesophageal injury in 8% of patients. These figures are comparable to the 8.7% (2 patients of the 23) of patients with oesophageal injury that was recorded in a study conducted in a tertiary hospital in the Western Cape province of South Africa^7^ which looked at patients with PNIs that were imaged primarily for vascular injuries as well as other neck injuries.

In line with previous studies, the current study confirmed PNIs and oesophageal injuries in mostly young males, who accounted for 88% and 83% of the injuries, respectively.^5,6,9,10^ This is likely because mostly young males are involved in crime, with high numbers of male-on-male violence.^5^ The most common mechanism of injury to the neck was secondary to stab wounds (57/75 [75%]), as has been reported in other studies. An audit on PNIs performed in KwaZulu-Natal found that 89% of injuries were secondary to stab wounds.^9^

In this study odynophagia was the symptom with the greatest frequency (33%), followed by dysphagia (17%). These findings support current literature where dysphagia and odynophagia have been described as strongly suggestive of oesophageal abnormality.^23^ Most PNIs were in Zone I in this study, although Zone II has been described as the most common site.^24^ Another South African study in which stab wounds were the predominant mechanism of injury also showed Zone I as the most common site of injury.^25^

The current study found that CTA was more sensitive for detecting oesophageal injury than fluoroscopic oesophagography, with a sensitivity of 100%. This is congruent with a prospective study that was performed at a London trauma centre looking at injuries in penetrating Zone II neck trauma, which also showed a sensitivity of 100%, with a single false positive.^24,26^ Their study, however, differed in that they used surgical exploration to confirm the presence of aerodigestive injuries. Inaba et al. conducted a multicentre prospective study looking at accuracy of CTA in evaluating injury after penetrating trauma, and also reported 100% sensitivity in identifying three oesophageal injuries. A combination of invasive diagnostic tests, surgical findings and clinical follow up were used as the reference standard.^11^ Gonzalez et al., however, showed a low sensitivity of 50% for CTA in assessing penetrating aerodigestive neck injury. Computed tomography angiography missed two of the four oesophageal injuries, both of which were detected at exploration.^26^ The article did not specify the criteria used for passing off a CTA as positive or negative and perhaps a difference in this definition may account for the discrepancy.

Computed tomography angiography fared poorly on specificity at 0% due to a high number of false positives. The cohort in the current study consisted of patients with suspected injury secondary to penetrating neck trauma, which meant patients were more likely to have concomitant injuries such as tracheal or laryngeal injuries, haemothoraces and pneumothoraces, all of which have overlapping radiological signs with oesophageal injury. This study also had no true negatives, further reducing the specificity. Only patients who were suspected to have digestive tract injury from clinical and CTA findings were sent for fluoroscopic examination. Numerous other studies have shown high specificity rates ranging from 76% to 100%.^24^

On fluoroscopic oesophagography the relationship between sensitivity and specificity was reversed with a high specificity and low sensitivity. Fluoroscopic oesophagogram was used as the confirmatory test to diagnose oesophageal injury. It has a less than 10% false negative rate.^18^ Ideally, if the water-soluble contrast is negative, it should be repeated with barium, which has a higher sensitivity for detecting perforation.^15^ All our studies were carried out with water-soluble contrast. Using water-soluble contrast only can have false negatives in the case of small perforations,^15^ and this may have contributed to underestimating the accuracy of our CTA.

Computed tomography angiography, however, remains attractive as it can be used to assess other neck structures and concomitant injuries.^23,27^ Haemothoraces, thyroid lobe injuries and associated mandibular fracture were present in the patients presenting with PNI. Of the patients with confirmed oesophageal injury, one had a haemothorax, one had vertebral artery injury, and there were several associated osseous injuries. This is comparable to an American study carried out at two hospitals (Ben Taub General and Jefferson Davis), which reviewed 77 patients with penetrating injuries of the oesophagus and looked at their surgical management. The study showed most associated injuries to be vascular, pulmonary and tracheal or laryngeal.^21^ The most seen CTA sign in patients with oesophageal injury was air in the deep spaces, followed by air in the superficial tissues, and pneumomediastinum. Madsen et al. evaluated patients with deep cervical and/or mediastinal emphysema on CTA and subsequently identified all oesophageal injuries in their study population.^24,25^

### Study limitations and suggestions

This was a retrospective study with inherent biases. A retrospectively analysis of the reports was conducted as part of patient investigation; no repeat image reading was performed. The sample size was limited as most of the patients with PNI do not have routine fluoroscopic oesophagography and not all reports were available as a result of archiving technicalities. Verification bias was unavoidable as all patients selected for study underwent both CTA and fluoroscopic oesophagography, with underrepresentation of true negatives.

There is a need for larger prospective studies to further define the role of CTA, especially in settings with limited resources. Some studies have shown that CT oesophagography, involving the administration of oral contrast during the scan to opacify the oesophagus, is potentially comparable to or even outperforms fluoroscopic oesophagography. Combining these two approaches could offer an effective strategy for minimising delays in time and costs. Conducting a study that evaluates the integration of these imaging techniques could be beneficial in assessing and establishing an optimal imaging protocol.

## Conclusion

Although oesophageal injuries resulting from penetrating neck trauma are uncommon and pose diagnostic difficulties, they carry a significant risk of morbidity and mortality. Early and accurate diagnosis is crucial in improving clinical outcomes for patients afflicted with such injuries.

Computed tomography angiography demonstrates a high sensitivity for detecting oesophageal injuries and offers the convenience of being more accessible and readily obtainable. Additionally, it allows for the assessment of concomitant injuries, aiding in clinical decision-making. Despite these benefits, CTA exhibits limited specificity, and its overall diagnostic accuracy falls short of the superior accuracy provided by the gold standard of fluoroscopic oesophagography. Computed tomography angiography should therefore be used as a first line, adjunct imaging modality in the assessment of patients with suspected oesophageal injury. All patients with suspected oesophageal injury who have abnormal CTA findings should be referred for fluoroscopy.
